# Prader-Willi syndrome: A primer for clinicians

**DOI:** 10.1186/1687-9856-2011-12

**Published:** 2011-10-18

**Authors:** Mary Cataletto, Moris Angulo, Gila Hertz, Barbara Whitman

**Affiliations:** 1 The Prader-Willi Syndrome Center at Winthrop University Hospital, 120 Mineola Blvd.-Suite 210, Mineola, N.Y. 11501, USA; 2 Huntington Medical Group, PC, Sleep Disorders Center, 180 East Pulaski Rd., Huntington Station, N.Y. 11746, USA; 3 Saint Louis University School of Medicine, 1465 S. Grand, St. Louis, Mo. 63104, USA

## Abstract

The advent of sensitive genetic testing modalities for the diagnosis of Prader-Willi syndrome has helped to define not only the phenotypic features of the syndrome associated with the various genotypes but also to anticipate clinical and psychological problems that occur at each stage during the life span. With advances in hormone replacement therapy, particularly growth hormone children born in circumstances where therapy is available are expected to have an improved quality of life as compared to those born prior to growth hormone.

This manuscript was prepared as a primer for clinicians-to serve as a resource for those of you who care for children and adults with Prader-Willi syndrome on a daily basis in your practices. Appropriate and anticipatory interventions can make a difference.

## Introduction

First described by Prader, Labhart and Willi in 1956 [1], this syndrome represents the most common genetic cause of obesity with an estimated incidence of 1:15,000 to 1:25,000 live births [2,3]. Reported prevalence rates vary among countries but both sexes appear to be equally affected. Prader-Willi syndrome (PWS) is the first human syndrome identified with genomic imprinting [4]. The original descriptions of this syndrome included short stature, hypotonia, hypogonadism and mental retardation [1]. As infants grow to age 2-4 years, failure to thrive related, at least in part, to poor muscle tone and poor suck are replaced by increased appetite and food intake resulting in obesity and its comorbidities. Early diagnosis and intervention to prevent obesity and the associated complications are critical.

### Genetic testing and genetic counseling

Candidate genes for Prader-Willi syndrome have been located on the long arm of chromosome 15q11-q13. These genes are physiologically imprinted and silenced on the maternally inherited chromosome. PWS arises when the paternally derived genes are missing, defective or silenced. The frequencies of each are shown in Table 1.

**Table 1:** Frequency of genetic subtypes associated with PWS

**Subtype**	**Frequency**
Paternal deletion of chromosome 15q11-q13 (type I or II)	75%

Maternal uniparental disomy (UPD)	24%

Imprinting center defects (ID)	1%

Translocation	< 1%

High resolution chromosomal analysis (HRCA) is done along with the fluorescence *in situ *hybridization (FISH) to detect deletions and translocation of chromosome 15 [5]. Deletion has been divided in type I (TI) and II (TII) according to the size. Studies indicate that individuals with the TI (~500 kb larger than TII) generally have more behavioral and psychological problems than individuals with the TII and UPD [6]. Negative FISH or karyotype analysis does not exclude the diagnosis and thus if done first should be followed by DNA methylation analysis. DNA methylation analysis is the only technique which can both confirm and reject the diagnosis of PWS, and therefore should typically be the investigation of choice. This is most commonly done using DNA methylation-specific techniques at the ***SNURF-SNRPN ***locus [7,8]. If DNA methylation analysis shows only a maternal pattern, then PWS is confirmed. Further methods may then be performed to determine the genetic subtype and allow appropriate genetic counseling. DNA methylation analysis has a sensitivity exceeding 99%; however, it does not differentiate between deletion, UPD and imprinting defect. In order to distinguish a maternal UPD from an imprinting defect, further DNA polymorphism analysis should be performed on the proband and parents [9,10].

Most cases of Prader-Willi syndrome occur sporadically. The overall recurrence risk is dependent on the type of molecular defect. In families where the proband has either maternal disomy or deletion, the recurrence risk is small (less than 1%). Patients with an imprinting defect warrant further investigation in a specialized laboratory to determine whether an imprinting center deletion is present. Those families with a child with an imprinting center deletion have a recurrence risk of up to 50% if the father of the child is a carrier for the imprinting center deletion [11]. When a deletion is the result of a translocation or structural rearrangement involving chromosome 15, then the recurrence risk can be high. The actual risk in individual families depends upon the rearrangement which they carry. Overall, the risk of recurrence in the case of chromosomal translocations has been estimated up to 15%.

In the future the methylation-specific multiplex ligation PCR amplification may be more widely used because it has the advantage of combining dosing and DNA methylation analysis in one assay, thus distinguishing different subtypes [12].

### Clinical Presentation

**Figure 1:**
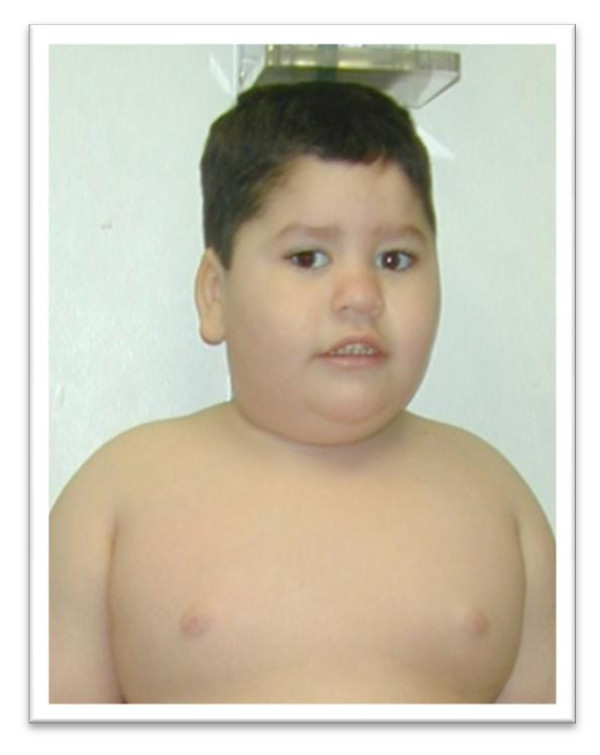
**Typical Facial Features of Child with Prader-Willi syndrome (Photograph with Permission)**.

**Figure 2:**
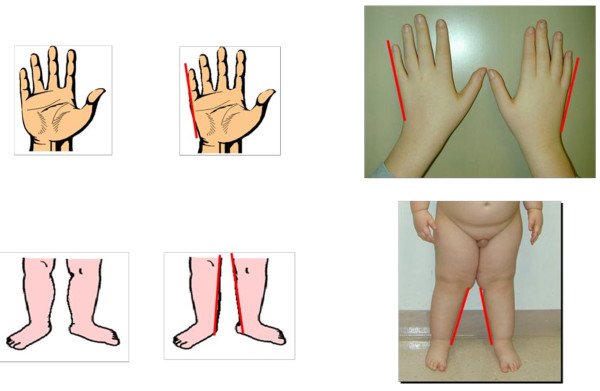
**Typical Findings of Hands and Legs in Individuals with Prader-Willi syndrome**.

Infants with Prader-Willi syndrome present with neonatal hypotonia, hypoplasia of the clitoris/labia minora in girls and small penis and undescended testis in boys. Their hypotonia is associated with poor suck and feeding, often resulting in failure to thrive. Mothers may report decreased fetal activity and infants are often found in the breech position at the time of delivery. Clinical features include increased neonatal head:chest circumference ratio, narrow bifrontal diameter, dolichocephaly, almond shaped eyes, downturned angles of the mouth with abundant and thick saliva, small hands and feet with straight borders of the ulnar side of the hands and inner side of the legs. The presence of some of these features associated with neonatal hypotonia should alert physicians for early diagnosis of PWS during infancy. These features may become more prominent by age 2-3 years (Figures 1 and 2). Excessive eating and obsession with food generally begins in the preschool age group and will lead to morbid obesity if not controlled.

As these individuals age, manifestations, such as obesity, short stature, hypogonadism, skin picking, learning disabilities, behavioral and psychiatric problems become more evident. Consensus criteria for the clinical diagnosis of PWS were first established in 1993 by Holm et al [13]. These criteria were used until the introduction of the highly sensitive genetic testing, described above. Currently, these criteria are used as a screening tool for determining the need for further PWS specific genetic testing. In many infants poor cry and unexplained hypotonia may be the only clear clinical manifestations and indication for genetic testing.

As many as 16.7% of patients diagnosed with molecular testing do not meet these clinical diagnostic criteria, therefore a revised clinical criteria to help identify the appropriate patients for DNA testing was proposed in 2001 [14] and modified in 2008 [15]. See Table 2 for composite, including additional features suggested by authors.

**Table 2:** Indications for DNA testing

Age at assessment	Features sufficient to prompt DNA testing
Birth to 2 yr	Hypotonia with poor suck
2-6 yr	Hypotonia with a history of poor suck
	Global developmental delay
	Short stature and/or growth failure associated with accelerated weight gain
6-12 yr	Hypotonia with a history of poor suck (hypotonia often persists)
	Global developmental delay
	Excessive eating (hyperphagia, obsession with food) with central obesity if uncontrolled
	Short stature and/or decreased growth velocity*
13 yr through adulthood	Cognitive impairment, usually mild mental retardation
	Excessive eating (hyperphagia, obsession with food) with central obesity if uncontrolled
	Short stature and/or decreased growth velocity*
	Hypothalamic hypogonadism and/or typical behavior problems (including temper tantrums and obsessive-compulsive features)

*Features added by the authors

## Endocrine Issues

### Hypogonadism

Hypothalamic and pituitary dysfunction is most commonly manifested as hypogonadism, obesity and short stature. Hypogonadism with genital hypoplasia (cryptorchidism, scrotal or clitoral hypoplasia) can be identified in the newborn period. Cryptorchidism is present up to 86% of boys from birth [16,17] Undescended testes should be treated within the first year of life. There is evidence that early damage to the germ cells that produce sperm begins at this age. Scrotal hypoplasia and small penis however can make orchiopexy and circumcision difficult in infants with PWS. Repeat surgical interventions are frequently required, especially in those infants with underdeveloped scrotal sacs.

The most effective treatment for undescended testes is surgery. The Committee on Genetics, American Academy of Pediatrics however, recommends a therapeutic trial of human chorionic gonadotropin (hCG) for treatment of undescended testes before surgery, because avoidance of general anesthesia is desirable for infants with low muscle tone and potential for underlying respiratory compromise [18]. The precise mechanism of action in regards to testicular descent is unknown but benefits of a course of hCG may include increased scrotal size and partial normalization of phallus length, thereby improving surgical outcomes for undescended testes and facilitating later standing micturition.

Premature adrenarche (PA) is the precocious appearance of pubic and/or axillary hair and less commonly an apocrine odor, comedones, and acne without other signs of puberty or virilization. PA is usually seen before age 8 and 9 years, in girls and boys respectively. PA has been reported in 57% of children receiving GH therapy [19] but in general, pubertal development in PWS is characterized by normal adrenarche, pubertal arrest, and hypogonadism due to variable combinations of a unique primary gonadal defect and hypothalamic dysfunction [20,21].

At some stage almost all subjects will require sex hormone replacement therapy. Mental retardation should not be a contraindication to allow normal pubertal development or preclude sex hormone replacement at any age in those affected individuals. Regardless of body weight, patients with PWS have increased body fat content. Those individuals with low body weight or significant low sex hormone levels during adolescence and adulthood should be considered for sex hormone treatment. There is no consensus as to the most appropriate regimen for sex hormone replacement therapy in PWS. Intramuscular testosterone is given every 3 -4 weeks. Testosterone gel preparations can be useful in selected cases, although precautions must be taken to avoid cross-contamination. Whatever preparation is preferred, the initial dose should be one third to one half of the normally recommended androgen dose to prevent the aggressive behavior occasionally seen in some individuals. In females with PWS, the use of gonadal hormone replacement should be considered if there is amenorrhea/oligomenorrhea or decreased bone mineral density (BMD) in the presence of reduced estradiol levels. Hypogonadism is a common but not necessarily universal finding in adults with PWS [16,17]. Sexual counseling and contraceptive treatment should be used as appropriate, especially in the presence of complete sexual maturation, including regular menses. There are a few case reports of pregnancy in females with PWS [22,23]. Their cognitive dysfunction, social and emotional immaturity and the risk of Angelman syndrome in offspring of PWS deletion mothers prompt us to advise against pregnancy. At present there are no reports of paternity in PWS. Estrogen and androgen status should be monitored yearly during adolescence and adulthood and BMD assessed as indicated by dual-energy x-ray photon absorptiometry.

### Adrenal insufficiency

When the pituitary begins to fail, there is generally a specific sequential failure of pituitary hormones, starting with growth hormone (GH), continuing through luteinizing (LH) and follicle stimulating hormone (FSH) deficiency, and culminating in the loss of thyrotropin stimulating hormone (TSH) and adrenocorticotropic hormone (ACTH). Generally ACTH is the last to be affected. Hypothalamic dysfunction is characteristic of individuals with PWS, therefore the clinical manifestations of pituitary hormone deficiency are expected. Short stature and hypogonadism, as a result of GH and gonadotropin (LH and FSH) deficiencies are seen in most individuals with this genetic syndrome. Under normal conditions the secretion of cortisol, the main adrenal glucocorticoid in humans is under the dominant control of pituitary ACTH. Clinical manifestations of adrenal insufficiency, however are uncommon in individuals with PWS.

The circadian peak serum cortisol usually occurs around 0800 hours. An extremely low basal serum cortisol at this time, below 100 nmol/liter (3.62 mcg/dl), may be assumed to demonstrate true cortisol deficiency [24]. However, levels at other times have little diagnostic utility. For this reason, various dynamic tests including insulin tolerance test (ITT), ACTH stimulation and metyrapone test have been devised to assess whether the patient can provide a stress-induced rise in cortisol similar to a normal person. The insulin tolerance test evaluates the integrity of the entire hypothalamic-pituitary axis (HPA) by inducing symptomatic and biochemical hypoglycemia, with cortisol then measured over 120 minutes. Peak cortisol values greater than 550 nmol/liter (19.75 mcg/dl) is considered a normal response. Under supervision by a nurse or physician, the ITT (0.15 U/kg administered intravenously (IV) is relatively safe [24]. The GH reserve can also be estimated with ITT.

Due to the relative inconvenience of the ITT, suggestions have been made to use a simpler and less invasive surrogate. The most widely performed is the short ACTH (Cortrosyn ™) stimulation test, where ACTH 0.25 mg is injected IV or IM and serum cortisol is measure at 0, 30 and 60 minutes. A peak cortisol is defined as normal if it is greater than 550 nmol/liter (19.75 mcg/dl) at any of these time points.

In 2008 a study using the overnight metyrapone test reported a 60% prevalence of central adrenal insufficiency (CAI) in children with PWS [25]. Based on the high prevalence of CAI, the authors suggested treatment with hydrocortisone during acute illness in patients with PWS unless CAI has recently been ruled out with a metyrapone test. ITT, as the gold standard dynamic test suggests that metyrapone test with an ACTH cut off of 33 pmol/l yields a high false positive rate. In our experience in the PWS center at Winthrop University Hospital, New York, we have not found any abnormal response to ITT or low cortisol levels during surgical stress of different natures. Three recent studies using a more sensitive stimulation and spontaneous acute stress in larger numbers of patients did not find high prevalence of central adrenal insufficiency in Prader-Willi syndrome [26-28]. Thus rather than common, CAI seems to be a rare event in children and adults with PWS, however, they should be evaluated and treated accordingly.

### Growth & Growth hormone deficiency

Length in newborns with PWS is normal but there is significant decrease in growth velocity after age 2-3 years with final adult height ~ 2 standard deviations (SD) below the mean for the normal population [29-31]. Only a small percentage of children with PWS are GH sufficient, thus provocative testing is not required in the face of reduced growth velocity.

Multiple studies have documented the benefits of GH therapy in individuals with PWS including, but not limited to, improvements in lean body mass, decreased body fat, increased bone mineral density, and normalization of adult height [19,32-37]. The benefits of starting GH treatment as early as age 2 years are well established, but there is increasing evidence of additional benefit to starting therapy between ages 6-12 months, particularly in terms of motor development, muscle, head circumference, and possibly cognition [35-38].

It should be stressed that GH therapy should be used in conjunction with appropriate nutritional intake and physical activity. GH treatment should not be viewed as a substitute for diet and exercise. Treatment should commence using standard dose guidelines (0.18-0.3 mg/kg/week), given as a daily subcutaneous injection with careful monitoring of clinical status at regular intervals. Careful history and assessment of nutritional status, scoliosis, respiratory and sleep abnormalities should be evaluated prior to and during GH therapy.

Recent studies indicate that adults with Prader-Willi syndrome may also benefit from GH replacement therapy, with improvements in body composition, bone mineral density, exercise capacity, quality of life and well-being [39-45]. Treatment doses are typically started at 0.2 mg/day and increased by 0.2 mg increments as necessary to maintain IGF-1 levels within the normal range for age and gender. At the present time, documentation of GH deficiency by provocative testing is required for adults with PWS to receive insurance authorization for GH treatment in the United States. These patients should be monitored with IGF-1, glucose, insulin, lipid profile, BMD and cardiac evaluation during GH treatment [46].

Central hypothyroidism as a result of hypothalamic dysfunction can also be seen in individuals with PWS. Periodic monitoring of thyroid function, fasting plasma glucose and insulin level is strongly recommended regardless of growth hormone therapy.

## Neurocognition and Behavior

Decreased intellectual functioning was among the four original defining characteristics of PWS [1]. Subsequent studies document a typical neurobehavioral profile that includes altered intellectual functioning and centrally driven maladaptive behaviors, including the hallmark hyperphagia that exists in the context of a more extensive food related behavioral constellation, an age related emotional and behavioral profile, altered sensory processing, social deficits and for many a predictable psychiatric profile [47-51].

### Intellectual Functioning

Following the original description, early studies of intellectual development documented a wide range of intellectual abilities, although most affected individuals tested in the borderline to mildly slow IQ ranges. As more sensitive genetic testing has become available, the population of individuals with PWS has become more clearly defined. Table 3 highlights studies of individuals with PWS who had genetic confirmation of their diagnosis, and who received age appropriate and properly administered cognitive testing, supplemented with measures of adaptive functioning.

**Table 3:** Intelligence Quotient (IQ)

				Intelligence Quotient			
				Degree of Mental Retardation (%)			
Investigator	Year	Number enrolled	Mean Age*	Normal -Borderline	Mild	Moderate	Severe
Einfeld^a ^[47]	1999	46	17.7	21.6	64.9	13.5	0
Gross-Tsur^b ^[51]	2001	18	14.3	73	27	0	0
Deschee-Maeker [52]	2002	55	14.1	25.4	27.3	40	7.3
Whittington [53]	2004	55	21.0	31	41.8	27.2	0
Copet [54]	2010	85	24.2	7	54	39	0
Roof [55]	2000	47	23.2	24	38	30	8

a. Only 1/2 of subjects genetically confirmed, most IQ's from records

b. Did not give a measure of adaptive functioning

*Age in Years

The Israeli data are notable for the number of individuals testing in a normal range, and represents a distribution of IQ scores that is quite different from the remaining four studies. The reasons for this are unclear. Setting aside the Israeli data and averaging across the remaining studies, all with approximately the same number of participants, Full Scale IQ ranges are as follows: ≥ 70 in 21%; mild cognitive impairment in 47%; moderate cognitive impairment in 32% and severe to profound cognitive impairment in 2%. An earlier report by Curf and Fryns [56] reported a greater proportion of subjects both in the > 70 and in the mildly impaired range, however their population included many subjects for whom no genetic testing was available and thus may have included individuals who did not have PWS.

Separate from the overall range of functioning among an affected population is the question of subtype differences in intellectual functioning. Such differences may be relevant in understanding the role of various genes in the overall clinical features and phenotype of this disorder. While most studies have not found significant subtype differences in overall IQ scores, at least 2 studies have reported a greater number of UPD subjects with normal IQ scores when compared to those with deletion [57-59]. Indeed Torrado et al reported that 61.5% of those with UPD had a Full scale IQ > 70, while only 10.5% of the subjects with deletion scored in that range. However, the mean age of Torrado's subject population was 4.09 years (range 12 days-17 years), so that the significance and overall stability of the obtained IQ scores is open to question. Statistically significant subtype differences have been reported for overall Verbal vs. Performance IQ scores with at least 2 studies reporting that those with UPD have higher verbal IQ scores and those with a deletion subtype have higher performance IQ scores [49,54], although more recently Copet et al [54] found that only the greater performance IQ of the deletion group vs a disomy group was statistically significant. Keep in mind that even when subtype scores are statistically significant, in no case have those differences ever reached the level of 1 SD for the test in question. Thus, whether these statistical differences are reflected as clinically relevant functional differences between subtypes is a question that must be raised.

In addition to mild cognitive deficits which are seen in most individuals with PWS, the overall cognitive profile at all ages includes cognitive rigidity, attentional deficits, problems with short term memory, auditory processing, sequential processing, arithmetic and social cognition. Relative strengths include long term memory, visual spatial performance, simultaneous processing, unusual abilities with jigsaw puzzles, particularly in the deletion subtype and for some reading decoding (devoid of comprehension).

### Neuro-behavioral Profile

While there are a number of clinical descriptions of a typical behavior profile among those with PWS, from the earliest efforts behavioral studies have primarily focused on describing and quantifying the development of problem behaviors and psychiatric difficulties. Despite calls to include investigations of strength and adaptive behaviors [60], these remain a rare study focus. Moreover many studies include such a wide age range, often including infants through late adulthood measured at a single point in time. Parceling out developmental aspects of the behavior profile requires a critical combination of clinical and empirical evidence. Nonetheless studies across time, taken together, yield a general behavior picture that is remarkably consistent across affected individuals, despite variation in severity and intensity across individuals and within the same individual across time. Foremost among these behaviors is the hyperphagia and associated food related behavior constellation. In addition, most clinical and empirical studies document the commonality of hoarding; cognitive rigidity along with the need for sameness, temper outbursts and emotional lability, repetitive and perseverative behaviors and skin-picking.

Hyperphagia remains the cardinal defining feature of PWS. Nonetheless, the hyperphagia is only one aspect of a larger food-related behavior constellation that included preoccupations surrounding food; food seeking/foraging; sneaking, hiding and hoarding food; eating unusual food-related items (sticks of butter, used cooking grease, decaying food, garbage), food flavored items, such as shampoos and for many, manipulative and sometimes illegal behaviors designed to acquire food. While hyperphagia is found in other genetic syndromes (e.g. WAGR syndrome, Bardet-Biedel syndrome), the development of the hyperphagia and eating patterns associated with PWS, distinguish the hyperphagia associated with PWS from other disorders. Primary among these is the relatively late age of emergence, the rapid escalation and intensification of the hyperphagia following several years of poor to relatively normal eating, often accompanied early on by failure to thrive. Additional distinguishing characteristics include the duration of eating, amount of food eaten and a delayed to absent deceleration of eating, leading to gorging when both physiologic satiation and volume induced discomfort should preclude additional intake.

In the daily run of life, this is reflected as constant talking about food and unrelenting requests and demands of parents and other caregivers for food, that when denied often precipitate a tantrum. This frequently happens at the grocery or while shopping in other stores that may also have food or candy aisles and at restaurants. The denial-related tantrums can be of such a nature that parents give in as a method of avoiding the behavior, thus creating a pattern that escalates in severity and intensity over time. In addition affected individuals display a constant preoccupation with food leading to extraordinary vigilance for detecting food anywhere in the environment often resulting in stealing other's lunches at school or work, food from teacher's desks or caregiver's purses, stealing food at home or in shopping areas, begging others for food, foraging in garbage cans, entering another's home in search of food and manipulating others to obtain food. It is a rare parent who has not received a call from the school or vocational site indicating that the affected individual has been obtaining extra food by convincing caregivers that parents are ill or haven't had time to feed them, often for an extended period of time.

The etiology of the hyperphagia remains elusive. Long attributed to a hypothalamically mediated failure of satiety control [61,62], current studies suggest a far more complex etiology than previously hypothesized, including, for many a theoretical reorientation that views the hyperphagia as reflecting a starvation syndrome rather than an obesity syndrome. From this vantage point, the obesity associated with PWS is seen as resulting from a physiologic signaling defect indicating that the body is in a constant state of starvation similar to that of malnourished infants, thus leading to the constant drive to obtain food.

To date there is no effective pharmacologic intervention. Management is environmental and behavioral, requiring restricted access to food in all environments, locks on cabinets and refrigerators, constant supervision, as well as measures to prevent obesity which include calorie restrictive diets, consistently scheduled meals and snacks and regularly scheduled physical activity. While simple in concept, the number of environments encountered in any given day, along with the cooperation needed from the individuals in those environments presents challenges that may be insurmountable for some families. Accounts from both parents and individuals with PWS support that strict limit setting with regard to foraging and food access is associated with reduced anxiety and a sense of safety [63].

### Behavioral Disturbances

Separate from the food related behavioral issues, multiple studies document that affected individuals are more prone to behavioral disturbances including hoarding; inflexibility of thinking and behavior; repetitive and perseverative behaviors; the need for sameness; tantrums and emotional lability; and skin picking [62]. Furthermore, the overall rate, severity and chronicity of these disturbances are frequently more intense than those associated with comparable genetic disorders or cognitive impairments or other obese groups [43,64,65]. Like the hyperphagia, the behavioral patterns appear to evolve over time with predictable epochs. Most authors agree that, on the whole, infants and young toddlers with PWS are affectionate, placid and generally cheerful, largely compliant and usually cooperative. However as the hyperphagia emerges, a separate and distinctly negative behavioral shift is also observed including an emergence and escalation of both food and non food related tantrums, a shorter tolerance for frustration combined with an overreaction to frustration; repetitive and ritualistic behavior as well as becoming " stuck " or perseverating on issues both in thought and speech; and other behavior problems including increasing oppositional tendencies, a lessened ability to " go with the flow " along with a drive for sameness and " increasing stubbornness and rigidity ". Comparison studies indicate that typically developing children and other children with mental retardation also exhibit the emergence of such behaviors, but they occur only transiently, that is, the problem appears and then subsides. However the emergence of such behaviors in those with PWS not only is persistent, but appears to escalate with age, increasing in severity and intensity, independent of intellectual, language or motor abilities [66,67].

Chronic behavior disturbances, including emotional lability accompanied by unbridled displays of temper; repetitive, ritualistic and compulsive like behaviors and hoarding become particularly prevalent in adolescence and persist well into adulthood, distinguishing these individuals from both younger children and older individuals with PWS, as well as from typical adolescents. In addition there has recently been an increasing recognition of accompanying social cognition and social interaction deficits among affected individuals, including an inability to read facial expressions of emotion and difficulty interpreting visually presented social information, such as those inherent in any social interaction [68,69]. Indeed several authors sum up the behavioral profile of those with PWS as " egocentric " and who argue, lie, manipulate and confabulate to change rules, obtain their wishes or justify behavior. Their social judgement is poor, even considering their intellectual ability; and interpretations of visually presented social information is on a level with children who have pervasive developmental disorder [62]. Although some behavioral modulation is often seen in later ages, nonetheless problematic behaviors still exceed those seen in other comparison groups.

The expression of this behavioral phenotype does appear to depend, at least to some degree, on genetic subtype, with hoarding and overt behavioral expressions of frustration, anger and aggression more common among those with a deletion, as is a greater likelihood of modulation in middle adulthood. Internalizing and autistic spectrum behaviors are more common among those with UPD, and appear to be unremitting with little age related modulation [70,71].

Sensory Issues in the form of an altered sensitivity to pain, failure to exhibit fevers when expected and high rates of skin picking and gouging other body areas are extremely problematic among this group of individuals. While little research has been done around the issues of pain and lack of fever when expected, nonetheless blunted pain sensitivity and lack of appropriate fever response and the inherent dangers these present are clinically well documented. In this same spectrum, skin picking and other similar self injurious behavior occurs with increased prevalence in PWS when compared to a general intellectually impaired population [67,72]. When looking specifically at a population of those with PWS, skin picking is ubiquitous and when quantified, is as prevalent and problematic and in some studies even more so than hyperphagia [47]. It is the source of significant behavior and medical concerns and management challenges. Management is directed towards minimizing both the occurrence and impact of the behavior. To this end, a recent survey of 67 affected children and adolescents documented skin picking in 96% of respondents, which were directly associated with measures of anxiety, inattention, oppositional behaviors, function and quality of life [73]. Thus separate from medical management, behavior management must be focused on decreasing anxiety and boredom while eliminating opportunities for picking.

A number of case series across time have alluded to a small subset of individuals for whom seizures were problematic; however, it was generally thought that these represented incidental findings rather than a risk associated with PWS. A recent report by Fan et al [74] documented seizure activity in 10 of 56 subjects between the ages of 1-37 years, with suspicion in yet another 6 subjects. Among the ten subjects with documented seizure, one youngster's seizure disorder was attributed to sequelae of a grade II intraventricular hemorrhage associated with an early pre-term birth. Among the other nine cases, eight occurred in those with a deletion subtype and the other in a subject whose etiology was a presumed imprinting center defect; none were found among those with a disomy. After reviewing prior studies in which seizures were reported, the authors conclude that the overall prevalence of seizures in PWS is 16 -17%. Further they suggest that among those with a deletion, the risk for seizure in a PWS population is three to four fold times greater than that expected in a general pediatric population.

### Psychiatric Illness

For many, this wide ranging problematic, behavioral profile can become sufficiently impairing that hospitalization is needed, while for others it evolves into frank psychiatric difficulties. In fact, Cassidy found behavioral concerns to be the most frequent cause of hospitalization [75]. By late adolescence 15-17% will evidence a diagnosable mood disorder [76]. This appears to be especially true for those with UPD. Separate from a categorical psychiatric diagnosis, studies consistently document that the level of behavioral and thought psychopathology, such as delusions, paranoid ideation, common in adolescents and adults with PWS exceeds that of others with an intellectual disability of other origins or of a typical population [65,67], and is the primary source of residential and vocational failure and family stress among affected adolescents and adults. While pharmacologic intervention can be helpful and in the case of psychosis is mandatory, environmental restructuring and positive behavior support programs are even more critical for facilitating recovery and preventing further difficulties.

The proliferation of less invasive and more available brain imaging techniques during the past decade offers the possibility of new insights into the central origin of the behavioral picture associated with PWS. Mantoulan [77] compared MRI and PET scans in PWS and non-PWS individuals. MRI images did not show evidence of anatomic abnormalities. However the PET scans showed hypoperfused brain regions, particularly in the anterior cingulum and superior temporal regions. The authors went on to correlate regional cerebral blood flow (rCBF) in the hypoperfused regions with results from the Child Behavior Check list (CBCL) and identified significant correlations, which suggested that the functional consequences of these perfusion abnormalities in specific brain regions might help to explain the social and behavioral issues observed in PWS. Similarly, a number of studies looking at brain processing of food related concerns have yielded mixed findings [78-80]. Functional findings must be considered tentative as the technology is sufficiently challenging that few affected individuals can tolerate the technology nor cooperate with the necessary tasks. Nonetheless, as the technology evolves, the possibility for future studies holds great promise.

## Sleep Disturbance

### General Sleep Disturbances

Sleep disturbance is frequent in all patients with Prader-Willi syndrome independent of age and weight. PWS patients with normal weight have been shown to have multiple sleep disturbances including daytime sleepiness, disrupted sleep organization, prolonged nocturnal sleep and sleep disordered breathing (SDB). In infants, SDB consists primarily of central apneas and absent, reduced or delayed ventilatory responses and arousal to hypoxia and hypercapnia [81-84]. Adult individuals with PWS-related morbid obesity may have the preceding sleep disturbances as well as an obesity-hypoventilation syndrome and obstructive sleep apnea.

### Clinical Features

Abnormal sleep-wake organization, daytime sleepiness and sleep disordered breathing are the most common sleep related complaints. Irregular REM cycle and sleep disordered breathing appear as early as during infancy [85-87].

### Sleep wake organization

Early surveys of sleep in PWS reported long nocturnal sleep (> 8 hours) as a common finding [88]. Early morning awakenings and sleep fragmentation have also been reported [89].

The most consistent finding found in polysomnographic studies has been altered rhythm of REM sleep. Studies have shown a tendency towards shorter REM latency, increased number of REM periods and shorter intervals between REM cycles. Total percentage of REM sleep appears to be normal [90-92]. REM sleep alterations appear to be unrelated to the patient genotype [93].

### Excessive daytime sleepiness

Excessive daytime sleepiness (EDS) is an almost universal characteristic of individuals with PWS [88,89,94]. Clarke et al [88] reported EDS in more than 90% of their surveyed patients. Those patients who reported EDS were more likely to exhibit temper tantrums during the day.

Early studies, using daytime polysomnographic recordings, confirmed the presence of pathologic sleepiness in > 95% of the patients studied [95]. Later studies employed the multiple sleep latency test (MSLT). This test, also called a " nap test " is used to measure the time elapsed to sleep onset. It consists of 4 or 5 nap opportunities during the day. The MSLT is the gold standard to quantify sleepiness and diagnose disorders of excessive sleepiness. Studies with MSLT in individuals with PWS have shown abnormally short sleep latencies and frequent sleep onset REM periods (SOREMPs) [90,92,96]. Daytime sleepiness, as measured by MSLT, appears to be independent of the degree of sleep related breathing disorders [90,92,96-98], additionally suggesting that daytime sleepiness reflects a central, possibly hypothalamic hypoarousal.

EDS and the atypical REM sleep findings bear resemblance to features of narcolepsy. Indeed, preliminary evidence in a small number of patients showed that hypocretin deficiency, a characteristic finding in narcolepsy, was also found in individuals with PWS who were severely sleepy [99]. However, in a postmortem study, there was no significant difference in the number of hypothalamic hypocretin containing neurons between patients with PWS and age matched controls [100].

Several preliminary studies have suggested a link between EDS and disruptive behavior in PWS. Hertz et al [101] reported a significant correlation between daytime sleepiness and disruptive behavior as measured by care taker's ranking. Similarly, Richdale [89] reported increased behavioral disturbance in children and adolescents with PWS who also reported EDS. Finally, in Clarke et al's [88] survey, adult patients who reported EDS were more likely to exhibit temper tantrums during the day.

In contrast, Maas et al [94], reported no significant correlation between sleep disturbance and behavioral disturbance in a group of adults with PWS.

### Sleep Disordered Breathing (SDB)

Infants with Prader-Willi syndrome, as young as 4 months old, already demonstrate evidence of sleep disordered breathing. The most frequent type of SDB in infants with PWS are central apneas and periodic breathing [85,86]. Hypotonia and central control abnormalities likely play an important role. As obesity develops, around age 2 years, sleep apnea of the obstructive type becomes more common. Obstructive sleep apnea in both children and adults is directly associated with the degree of obesity and is inversely associated with age [97]. Oxygen desaturation is commonly seen even when the Apnea-hypopnea index (AHI) is only mildly elevated. The degree of sleep related oxygen desaturation may be severe, especially during REM sleep related hypotonia. Its severity is significantly increased with greater body mass index (91).

### Management of sleep disorders

A sleep evaluation of all patients with Prader-Willi syndrome should be routinely considered because of the high prevalence of sleep disturbances. Patients who are habitual snorers and/or sleepy during the day may require a polysomnogram to rule out sleep disordered breathing.

In recent years, as more patients are treated with growth hormone (GH), there has been a growing concern over the potentially adverse effects on sleep related breathing. GH may exacerbate OSA in PWS, especially in the presence of other respiratory complications [87]. Review of the death records from the French database of patients with PWS showed an association with respiratory tract infections in both GH and non GH treated patients, highlighting the need for added vigilance during these periods. In patients who were receiving GH treatment the concern for an adverse outcome of SDB and respiratory tract infection is particularly salient during the first nine months of treatment, more so among males [102]. Therefore, an overnight sleep study is recommended before GH therapy is instituted to rule out sleep disordered breathing.

The gold standard for the treatment of sleep apnea in adults is Continuous Positive Airways Pressure (CPAP) or BiPAP. In children adenoidectomy, tonsillectomy or adenotonsillectomy is often first line of treatment. Supplemental oxygen therapy may be added in the presence of obesity hypoventilation syndrome. The management of other sleep disturbances may include implementation of adequate sleep hygiene, sleep wake schedule regulation and even circadian rhythm modification.

In patients who present with excessive daytime sleepiness, a Multiple Sleep Latency Test (MSLT), is also indicated. Once diagnosed, daytime sleepiness can be managed pharmacologically or with behavioral intervention. The pharmacological management of daytime sleepiness has been controversial because of the potential side effects of stimulant medication. Additional research is needed to assess the effects of stimulant medication on daytime alertness, disruptive behavior and the general well being of the sleepy patient with PWS. Behavioral management of EDS focuses on improving nighttime sleep and scheduling daytime naps when needed.

## Gastrointestinal Issues

Abnormal surges in ghrelin may precede the characteristic hyperphagia seen in PWS. Whether this causes or is the result of the lack of satiety in PWS is not clear. Left unchecked, lack of appetite control can lead to morbid obesity. Low calorie and well balanced diets with rigorous supervision and restriction of food access combined with regularly scheduled meals and activities are recommended [15]. Reduced energy requirements have been reported for children with PWS as compared to healthy, age matched controls [103-105]. Those initiating growth hormone replacement therapy may require increased caloric load during the initial muscle building phase, but once lean mass accretion has stabilized, a reduced caloric limit may again be needed.

Poor oromotor control, muscle hypotonia and voracious eating with a limited time for mastication of food may lead to choking episodes. Choking accounts for approximately 8% of all PWS deaths. Binge eating has been seen in both obese and lean individuals with PWS. Acute gastric distention with necrosis and death has been reported with and without binging behavior. While acute gastric distention is frequently accompanied by vomiting in the general population, individuals with PWS have a decreased ability to vomit and may be missed due to lack of this important finding. These issues may be further complicated by their increased tolerance to pain which may be in part responsible for delays in seeking medical attention related to these episodes.

## Musculoskeletal Issues

The prevalence of scoliosis in PWS is high (30% before 10 yr of age; 80% after age 10 years) [106-109]. Many patients shows progression of scoliosis with age irrespective of the use of GH and therefore scoliosis should no longer be considered a contraindication for GH treatment in children with PWS.

Most published reports of scoliosis in children with PWS have been retrospective. Recent evaluation of concerns regarding worsening of scoliosis in patients currently receiving GH have not been substantiated [110,111]. Prospective studies however are warranted. Studies by Shim et al [111] showed a high prevalence of spinal deformity, limb malalignment and foot abnormalities. This group found correlations between various musculoskeletal abnormalities, independent of obesity, but noted that obesity may conceal some of these abnormalities, especially in the early stage. At this time annual musculoskeletal evaluations are recommended for scoliosis, hip dysplasia, foot abnormalities and lower limb malalignments [112].

Slipped femoral capital epiphysis is seen with increased frequency in otherwise healthy, obese children. This has not been reported with increased frequency in children with PWS.

Early work [113] compared gait strategies in patients with PWS with those of both obese and non obese healthy patients. Adults with PWS in their study were found to walk slower, with shorter stride length, lower cadence and longer stance phases compared to non PWS controls. Range of motion at the level of the knee and ankle and plantar-flexor activity were significantly reduced. Spatio-temporal gait parameters in adults with PWS were further evaluated. Using 3 D gait analysis in an attempt to develop rehabilitation therapies, Cimolin et al [113] found that participating adults with PWS showed cautious abnormal gait strategies characterized by longer stance duration, reduced anterior step length and lower velocity of progression. Hip flexion with a forward pelvic tilt was present throughout the gait cycle. Investigators felt that this reflected an attempt to achieve balance and stability in the face of excessive body weight.

## Prognosis

While there is no cure for Prader-Willi syndrome, major strides to improve quality of life have been made since the introduction of more sensitive genetic testing modalities which has allowed early diagnosis and intervention. The early use of GH has improved final adult height, body composition and muscle strength. Obesity and the consequences of obesity continue to be major risk factors for mortality in persons with PWS, even after correction for the effect associated with intellectual disability [114].

### Consent

Written informed consent was obtained from the parent/guardian of the patient for publication of the accompanying image.

## Competing interests

The authors declare that they have no competing interests.

## Authors' contributions

All authors contributed to the development and writing of this manuscript and each has many years of clinical experience in the care of individuals with Prader-Willi syndrome. All authors read and approved the final manuscript.
